# The Mast Cell Is an Early Activator of Lipopolysaccharide-Induced Neuroinflammation and Blood-Brain Barrier Dysfunction in the Hippocampus

**DOI:** 10.1155/2020/8098439

**Published:** 2020-02-24

**Authors:** Yiwei Wang, Huanhuan Sha, Leting Zhou, Yinan Chen, Qin Zhou, Hongquan Dong, Yanning Qian

**Affiliations:** ^1^Wuxi People's Hospital Affiliated to Nanjing Medical University, Wuxi, Jiangsu, China; ^2^The First Affiliated Hospital of Nanjing Medical University, Nanjing, Jiangsu, China; ^3^Jiangsu Cancer Hospital, Nanjing, Jiangsu, China

## Abstract

Neuroinflammation contributes to or even causes central nervous system (CNS) diseases, and its regulation is thus crucial for brain disorders. Mast cells (MCs) and microglia, two resident immune cells in the brain, together with astrocytes, play critical roles in the progression of neuroinflammation-related diseases. MCs have been demonstrated as one of the fastest responders, and they release prestored and newly synthesized mediators including histamine, *β*-tryptase, and heparin. However, temporal changes in MC activation in this inflammation process remain unclear. This study demonstrated that MC activation began at 2 h and peaked at 4 h after lipopolysaccharide (LPS) administration. The number of activated MCs remained elevated until 24 h after LPS administration. In addition, the levels of histamine and *β*-tryptase in the hippocampus markedly and rapidly increased within 6 h and remained higher than the baseline level within 24 h after LPS challenge. Furthermore, mast cell-deficient Kit^W-sh/W-sh^ mice were used to investigate the effects of MCs on microglial and astrocytic activation and blood-brain barrier (BBB) permeability at 4 h after LPS stimulation. Notably, LPS-induced proinflammatory cytokine secretion, microglial activation, and BBB damage were inhibited in Kit^W-sh/W-sh^ mice. However, no detectable astrocytic changes were found in WT and Kit^W-sh/W-sh^ mice at 4 h after LPS stimulation. Our findings indicate that MC activation precedes CNS inflammation and suggest that MCs are among the earliest participants in the neuroinflammation-initiating events.

## 1. Introduction

The incidence of central nervous system (CNS) diseases is rapidly increasing worldwide, and neurological disorders are a significant global socioeconomic burden [1]. The mechanisms leading to CNS diseases are complex and are related to the brain function state and the insult severity. Although the neuroinflammatory response may be not an initiating factor in CNS diseases, emerging evidence indicates that sustained neuroinflammation contributes to the progression of CNS diseases [2], such as neurodegeneration, brain injury, and ischemic stroke, and is a crucial target for therapeutic strategies [3–5].

Previous studies on neuroinflammation focus predominately on microglia and astrocytes, proinflammatory factor expression, and peripheral inflammatory cell infiltration [6]. Accumulating evidence has demonstrated that mast cells (MCs), known as mastocytes or labrocytes, are also implicated in immunity and inflammation [7, 8]. MCs in the CNS are typically found along cerebral blood vessels on the brain side of the blood-brain barrier (BBB). They are highly plastic cells derived from hematopoietic stem cells, and their number and degranulation can dramatically change depending on the environmental stimuli and activation states [9].

Accumulating evidence suggests that MCs are implicated in the pathogenesis of CNS disorders. Our recent studies have highlighted the role of MCs in brain neuroinflammatory responses, and our findings indicate that significant elevations of MC number and degranulation are associated with postoperative cognitive dysfunction through microglial activation [10]. In addition, altered MC degranulation has also been found in other CNS disorders, such as stroke [11], Parkinson's disease [12], and multiple sclerosis [13]. Microscopic investigations have also demonstrated infiltrated *β*-tryptase-containing MCs in human brains with amyloid deposits [14]. Further, MCs are powerful and fast sensors of brain injury [15–17] by releasing cytoplasmic secretory granules filled with immune and inflammatory mediators including histamine, *β*-tryptase, tumor necrosis factor-alpha (TNF-*α*), and interleukin- (IL-) 1*β* [18]. MCs also express receptors and ligands of various inflammatory pathways, including protease-activated receptor-2- (PAR2-) MAPK-NF-kappa B, histamine receptor 1 (H1R)/H4R-MAPK, and PI3K/AKT-NF-kappa B pathways [19, 20].

Microglia and astrocytes are known to be activated by signaling pathways or proinflammatory mediators from immune cells. Accumulating evidence has indicated that MCs are the early responders in brain injury and precede glial activation [8]. MCs may act as catalysts and amplifiers of neuroinflammatory processes and activate microglia and astrocytes, thus leading to the production of neurotoxic mediators. Therefore, MCs may play an essential role in neuroinflammation, and the role of MCs in neuroinflammation needs to be examined extensively.

Peripheral lipopolysaccharide (LPS, an endotoxin isolated from bacteria) challenge can elicit liable and persistent CNS inflammation [21, 22]. LPS is therefore frequently used to study neuroinflammation. Therefore, LPS is frequently used to study neuroinflammation. In this study, we investigated temporal changes in MC responses after LPS stimulation to elucidate the direct effect of MCs on neuroinflammation using wild-type and mast cell-deficient Kit^W-sh/W-sh^ mice.

## 2. Materials and Methods

### 2.1. Animals

Male Sprague-Dawley (SD) rats (weight, 250-300 g) and mast cell-deficient Kit^W-sh/W-sh^ mice (STOCK KitW-sh/HNihrJaeBsmJNju mice, 6 months old) and their littermate controls (C57BL6/J, 6 months old) were purchased from Mode Animal Research Center of Nanjing University (Nanjing, China). The SD rats were used in the first experiment to determine the best time point of MC activation, while the mast cell-deficient Kit^W-sh/W-sh^ mice and C57BL6/J mice were used to investigate the role of MCs in LPS-induced neuroinflammation. The animals were housed in plastic cages (five animals per cage) at the animal center of the First Affiliated Hospital of Nanjing Medical University in a standard 12 h light/dark cycle at a constant room temperature of 22.0 ± 1.0°C. The animals were allowed for free access to tap water and food. All experimental protocols and the animal care were approved by the Nanjing Medical University Animal Care and Use Committee (IACUC-14030126). All efforts were made to reduce animal use and suffering in this study in accordance with the National Institutes of Health (NIH) Guide for the Care and Use of Laboratory Animals (NIH Publications No. 8023, revised 1978).

### 2.2. Reagents and Antibodies

LPS (from Escherichia coli, 0111:B4) was purchased from Sigma-Aldrich (St. Louis, MO, USA). Antibodies against ionized calcium binding adaptor molecule 1 (Iba-1) and *β*-tryptase were purchased from Wako (Osaka, Japan). Antibodies against occludin and Fluoroshield Mounting Medium with 4′,6-diamidino-2-phenylindole were purchased from Abcam (Hong Kong, China). Antibodies against albumin and glial fibrillary acidic protein (GFAP) and goat anti-mouse and anti-rabbit secondary antibodies were obtained from Cell Signaling Technology (Beverly, MA, USA). Claudin-5 antibodies were purchased from Invitrogen (Carlsbad, USA). Mouse interleukin-1*β* (IL-1*β*), IL-6, IL-4, IL-5, and tumor necrosis factor- (TNF-) *α* enzyme-linked immunosorbent assay (ELISA) kits were obtained from eBioscience (San Diego, USA). Rat histamine ELISA Kit was purchased from BioVision (Santa Cruz, CA, USA). Radioimmunoprecipitation assay (RIPA) buffer and BCA kit were obtained from Beyotime (Shanghai, China).

### 2.3. Drug Administration

In the first experiment, male SD rats were assigned to either the LPS treatment group or the 0.9% saline control group (*n* = 6 rats per group). Rats in the LPS group received an intraperitoneal (ip) injection of LPS (2 mg/kg) diluted in saline solution (400 *μ*g LPS in 1 ml saline), and rats in the control group received only the saline solution. This dosage of LPS has been demonstrated to induce neuroinflammation in rats without impairing motor function [23].

In the second experiment, male C57BL6/J and Kit^W-sh/W-sh^ mice were administered intraperitoneally with a single dose of LPS (4 mg/kg) diluted in saline solution (80 *μ*g LPS in 1 ml saline). This dose of LPS has been demonstrated to elicit moderate brain inflammation according to body surface area conversion equation [24, 25] without compromising the survival (LD50 value of LPS in mice, 20 mg/kg) [26]. The mice were divided into the following four treatment groups: wild-type (WT)/control group (WT mice+saline, *n* = 6); WT/LPS group (WT mice+LPS ip-injection, *n* = 6); Kit^W-sh/W-sh^/control group (Kit^W-sh/W-sh^mice+saline, *n* = 6); and Kit^W-sh/W-sh^/LPS group (Kit^W-sh/W-sh^ mice+LPS, *n* = 6). No animals died or needed to be terminated because of LPS injection in this study.

### 2.4. Enzyme-Linked Immunosorbent Assay (ELISA)

The animals were rapidly transcardially perfused with ice-cold phosphate-buffered saline, and hippocampal tissues were removed and divided. The samples were centrifuged for 20 min at 12,000 × *g* at 4°C, and the supernatants were stored at –20°C until analysis. The levels of MC mediators, including histamine, TNF-*α*, and IL-1*β*, in the brain tissue extracts were measured with ELISA kits according to the manufacturer's instructions. Briefly, the supernatants were added in a 96-well microplate with primary antibodies and incubated at 37°C for 30 min. Subsequently, secondary antibodies conjugated with peroxidase were added and incubated for 30 min. The concentrations were spectrometrically determined by a micro ELISA reader.

### 2.5. Western Blotting

Proteins in hippocampal tissues were extracted in RIPA lysis buffer containing 50 mM Tris, 150 mM sodium chloride, 1% Triton X-100, 2 mM ethylenediaminetetraacetic acid, 1.5 *μ*g/ml leupeptin, and 1 mM phenylmethylsulfonyl fluoride, followed by centrifugation at 12,000 × *g* for 20 min at 4°C. The protein concentration was determined by BCA assay (Thermo Fisher Scientific, Waltham, MA). The supernatants were mixed with sodium dodecyl sulfate (SDS) sample buffer and were then heated at 100°C for 5 min. Equivalents of 20 *μ*g of extracted proteins were electrophoresed in 10% or 12% SDS-polyacrylamide gels and then transferred onto polyvinylidene difluoride membranes (Millipore, Bedford, MA). The membranes were blocked with 5% nonfat milk in Tris-buffered saline with tween 20 (TBST) and rotated for 1 h. Subsequently, the blocked membranes were incubated in specific primary antibodies diluted in 5% nonfat milk overnight (*β*-tryptase—1 : 500; IBA-1—1 : 200; GFAP—1 : 200; albumin—1 : 500; occludin—1 : 200; claudin-5—1 : 100). The membranes were then washed with TBST and incubated with anti-rabbit or anti-mouse IgG-HRP secondary antibodies for 1 h at 20°C. The ECL method was conducted with the Image Lab software (Bio-Rad, Richmond, CA). The protein amount was estimated by quantifying the intensity of protein bands using Image J (NIH, Bethesda, MD).

### 2.6. Immunohistochemistry

Briefly, animals were perfused with 0.01 M phosphate-buffered saline (PBS) followed by ice-cold 4% paraformaldehyde (PFA). The brains were removed and fixed in 4% PFA at 4°C overnight. Coronal hippocampal sections were cut at 40 *μ*M thickness using a cryostat (Leica Microsciences, Mannheim, Germany). The sections were then blocked with 3% horse serum (Invitrogen, New Zealand) containing 0.05% Triton X-100 for 1 h. Subsequently, the sections were incubated with specific primary antibodies at an indicated dilution based on the manufacturer's instructions at 4°C overnight (*β*-tryptase—1 : 200; IBA-1—1 : 100; GFAP—1 : 100; albumin—1 : 200; Avidin—1 : 100). After washing with PBS three times, sections were incubated with Alexa Fluor-conjugated secondary antibodies at room temperature for 2 h. Images were captured using a confocal microscope (Zeiss LSM 510; Zeiss, Oberkochen, Germany). Fluorescence intensity was analyzed with ImageJ and normalized to the fluorescence levels observed in untreated samples.

### 2.7. MC Quantification

We used the following two staining techniques to identify MCs: Alcian blue-safranin staining and Avidin-staining.

In Alcian blue-safranin staining, deparaffinized and rehydrated hippocampus sections were stained with 0.5% Alcian blue in 0.3% acetic acid, rinsed in water, and incubated for 30 minutes with 0.1% safranin in 1% acetic acid. In formalin-fixed brain sections stained with Alcian blue-safranin, the populations of activated MCs are designated as “mixed” (activated MCs staining red and blue [Alcian blue-safranin-positive]) [27].

In addition, immunohistochemistry was also used to stain MCs with fluorophore-conjugated egg white Avidin, which binds to heparin, an MC-specific glycosaminoglycan. Thus, the conjugated Avidin staining technique is a specific and reliable method for identifying activated MCs [16].

MC quantification was always performed in a blinded fashion by an experimenter that was unaware of the sample identity. The entire surface area of the CA1 of the hippocampus was scanned manually using a light microscope (Leica 2500), and the activated MCs were calculated using the Cell D software (Olympus).

### 2.8. Evan's Blue (EB) Extravasation

EB dye (2%) was diluted freshly in 0.9% saline before the experiment, and the diluted EB dye was administered via the right femoral vein at a dose of 5 ml/kg 30 min before perfusion [28]. The animals were then perfused with 0.9% saline transcardially for 20 min to remove the intravascular dye. Subsequently, each hemisphere was excised, weighed, and homogenized in 0.5 ml of trichloroacetic acid (Sigma-Aldrich, 50% in saline). After centrifugation (10,000 × *g*, 10 min), the EB dye in the supernatant was measured using a spectrophotometer at 620 nm.

### 2.9. Statistical Analysis

Data were first tested for normality (Shapiro–Wilk test) and expressed as either the mean ± SEM (subjected to normal distribution) or median and interquartile range (not subjected to normal distribution). To test the homoscedasticity, Levene's test was applied. Multiple comparisons were conducted using an appropriate analysis of variance (for comparing preselected pairs: Sidak's multiple comparison test; for comparing the mean of each column with the mean of a control column: Dunnett's multiple comparisons test). The alpha level was set at *P* < 0.05.

## 3. Results

### 3.1. LPS Induces MC Degranulation in the Hippocampi in Rats

#### 3.1.1. Increased Number of MCs in the Hippocampi in Rats after LPS Treatment

**Figure 1:**
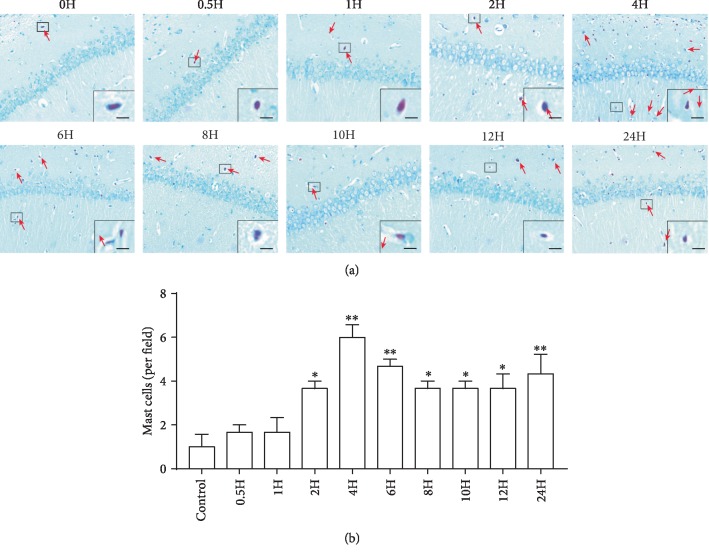
Alcian blue-safranin-stained mast cells in the hippocampi of rats after lipopolysaccharide (LPS) treatment. (a) Alcian blue-safranin staining was used to detect mast cells (arrow) in the CA1 of the hippocampus. Scale bar = 50 *μ*m. (b) Quantification of mast cells stained with Alcian blue-safranin. All experiments were repeated three times. ^∗^*P* < 0.05 and ^∗∗^*P* < 0.01 vs. controls. Data are presented as the mean ± SEM (*n* = 6).

**Figure 2:**
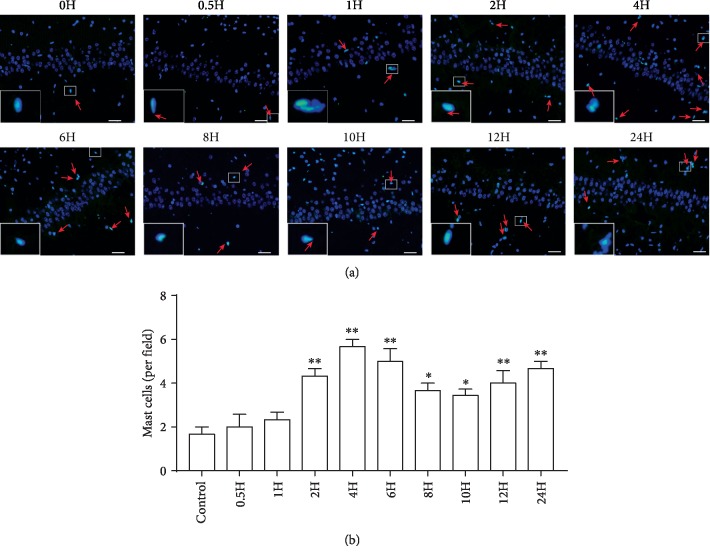
Avidin-stained mast cells in the hippocampi of rats after LPS treatment. (a) Avidin-positive cells (arrow) in the CA1 of the hippocampus. Scale bar = 50 *μ*m. (b) Quantification of mast cells stained with Avidin. All experiments were repeated three times. ^∗^*P* < 0.05 and ^∗∗^*P* < 0.01 vs. controls. Data are presented as the mean ± SEM (*n* = 6).

To investigate temporal changes of MC response to LPS, we analyzed the number of MCs in the hippocampal slides processed at 0, 0.5, 1, 2, 4, 6, 8, 10, 12, and 24 h after LPS treatment using the two methods mentioned above: Alcian blue-safranin staining (Figure 1) and Avidin staining (Figure 2).

The number of MCs differed among different time points (Alcian blue-safranin staining: *F*(9, 20) = 4.998, *P* = 0.0013; Avidin staining: *F*(9, 20) = 4.66, *P* = 0.002). The results consistently showed that the number of MCs began to increase at 2 h, peaked at 4 h, and remained elevated until 24 h after LPS administration (Figures 1 and 2).

#### 3.1.2. Increased *β*-Tryptase Expression after LPS Treatment

**Figure 3:**
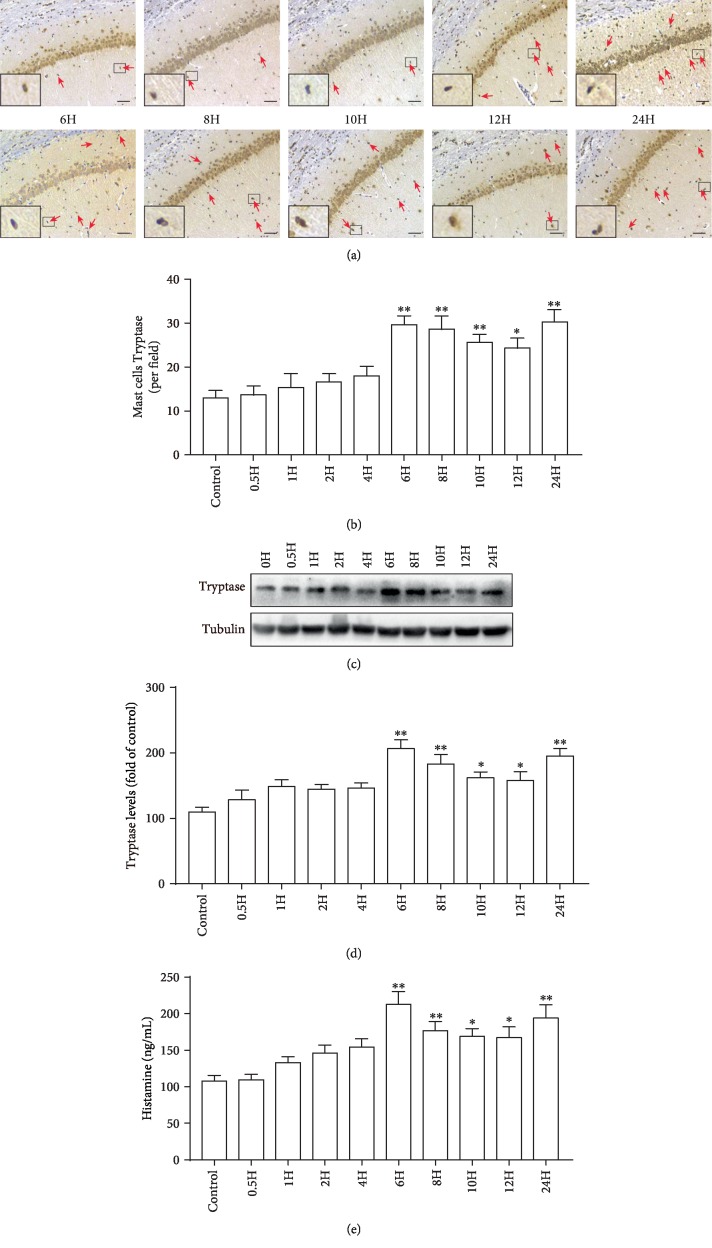
Changes in *β*-tryptase and histamine expression levels in the hippocampi of rats after LPS treatment. (a) Immunohistochemical detection of *β*-tryptase-positive cells (arrow) in the CA1 of the hippocampus. Scale bar = 100 *μ*m. (b) Quantification of *β*-tryptase-positive cells. (c) Protein levels of *β*-tryptase in the hippocampus were detected by western blotting. Each blot is representative of three experiments. (d) Expression levels of *β*-tryptase were quantified and normalized to Tubulin levels. Each value is expressed relative to that in the control group, which was set to 100. (e) Levels of histamine were detected by ELISA. ^∗^*P* < 0.05 and ^∗∗^*P* < 0.01 vs. controls. Data are presented as the mean ± SEM (*n* = 6).

*β*-Tryptase is the most abundant mediator stored in MC granules, and its release is a feature of MC activation [29]. To determine the effect of LPS on MC activation, we further evaluated the time-course changes in the expression of *β*-tryptase (0, 0.5, 1, 2, 4, 6, 8, 10, 12, and 24 h) using immunohistochemistry (Figures 3(a) and 3(b)) and western blotting (Figures 3(c) and 3(d)). As shown in Figures 3(a) and 3(b), the number of *β*-tryptase-positive cells increased steadily from 6 h and remained higher than the baseline level within 24 h after LPS stimulation by immunohistochemistry (*F*(9, 20) = 6.855, *P* = 0.0002). Similar results of *β*-tryptase protein levels were also observed by western blotting in Figures 3(c) and 3(d) (*F*(9, 20) = 5.099, *P* = 0.0012).

#### 3.1.3. Increased Histamine Expression after LPS Treatment

There are two main types of inflammatory mediators in MCs: preformed mediators and newly generated mediators. Preformed mediators such as histamine and neutral proteases (*β*-tryptase), which are stored in secretory granules, are secreted upon MC activation. Histamine is mainly derived from MCs and serves as a marker of MC activation [30]. ELISA analysis (Figure 3(e)) showed that the histamine level was low until 4 h but was then elevated from 6 h to 24 h after LPS treatment (0.5 mg/kg) in the hippocampi in rats (*F*(9, 20) = 6.255, *P* = 0.0003).

### 3.2. Attenuated Proinflammatory Cytokines and Increased Anti-Inflammatory Factors in the Hippocampi of Kit^W-sh/W-sh^ Mice after LPS Treatment

**Figure 4:**
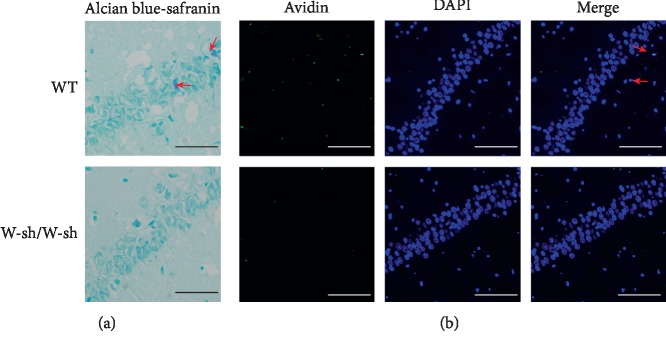
Alcian blue-safranin and Avidin staining of activated mast cells (arrow) in the hippocampi of WT and Kit^W-sh/W-sh^ mice (*n* = 4). Scale bar = 50 *μ*m.

We further evaluated the involvement of MCs in LPS-induced neuroinflammation using mast cell-deficient Kit^W-sh/W-sh^ mice. As shown in Figure 4, Alcian blue-safranin- and Avidin-stained MCs were found in the hippocampi in WT mice, but not in Kit^W-sh/W-sh^ mice, further indicating that these staining methods are specific techniques to identify MCs.

Our studies mentioned above demonstrated that the number of MCs started increasing within 2 h and peaked at about 4 h after LPS treatment. Therefore, the following experiments were performed at the time point of 4 h.

**Figure 5:**
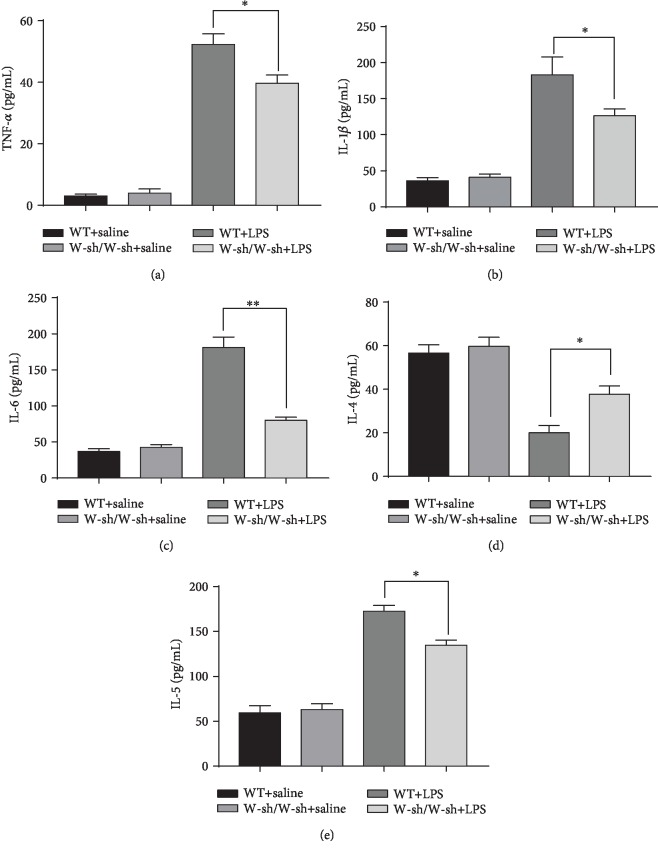
Cytokine changes after LPS treatment in the hippocampi of WT and Kit^W-sh/W-sh^ mice. Levels of tumor necrosis factor alpha (TNF-*α*) (a), interleukin 1 beta (IL-1*β*) (b), IL-6 (c), IL-4 (d), and IL-5 (e) were detected by enzyme-linked immunosorbent assay (ELISA). Data were representative of three independent experiments. ^∗^*P* < 0.05 and ^∗∗^*P* < 0.01 vs. the WT+LPS group. Data are presented as the mean ± SEM (*n* = 6).

It is widely recognized that LPS-induced neuroinflammation is mainly due to the excessive secretion of proinflammatory factors and the inhibition of anti-inflammatory factors; therefore, we further assessed the levels of TNF-*α* (Figure 5(a)), IL-1*β* (Figure 5(b)), IL-6 (Figure 5(c)), IL-4 (Figure 5(d)), and IL-5 (Figure 5(e)) using ELISA kits. As shown in Figures 5(a)–5(e), the expression of TNF-*α* (adjusted *P* = 0.9978), IL-1*β* (adjusted *P* = 0.9989), IL-6 (adjusted *P* = 0.9917), IL-4 (adjusted *P* = 0.9738), and IL-5 (adjusted *P* = 0.9921) was similar in saline-treated WT mice and saline-treated mast cell-deficient Kit^W-sh/W-sh^ mice. LPS treatment (4 h after injection) significantly increased TNF-*α* (WT mice: adjusted *P* < 0.0001; mast cell-deficient Kit^W-sh/W-sh^ mice: adjusted *P* = 0.0003), IL-1*β* (WT mice: adjusted *P* = 0.0003; mast cell-deficient Kit^W-sh/W-sh^ mice: adjusted *P* = 0.0094), IL-6 (WT mice: adjusted *P* = 0.0001; mast cell-deficient Kit^W-sh/W-sh^ mice: adjusted *P* = 0.0436), and IL-5 (WT mice: adjusted *P* = 0.0001; mast cell-deficient Kit^W-sh/W-sh^ mice: adjusted *P* = 0.003) levels in the hippocampi. However, the levels of TNF-*α* (adjusted *P* = 0.0482), IL-1*β* (adjusted *P* = 0.0427), IL-6 (adjusted *P* = 0.0047), and IL-5 (adjusted *P* = 0.0157) were significantly lower in LPS-treated mast cell-deficient Kit^W-sh/W-sh^ mice than in LPS-treated WT mice. Moreover, as a typical anti-inflammatory cytokine, the expression of IL-4 was greatly decreased after LPS administration (WT mice: adjusted *P* = 0.006; mast cell-deficient Kit^W-sh/W-sh^ mice: adjusted *P* = 0.0003). In contrast, IL-4 levels (adjusted *P* = 0.0471) were higher in LPS-treated mast cell-deficient Kit^W-sh/W-sh^ mice than in LPS-treated WT mice.

### 3.3. Inhibited LPS-Induced Microglial Activation in the Hippocampi of Kit^W-sh/W-sh^ Mice

**Figure 6:**
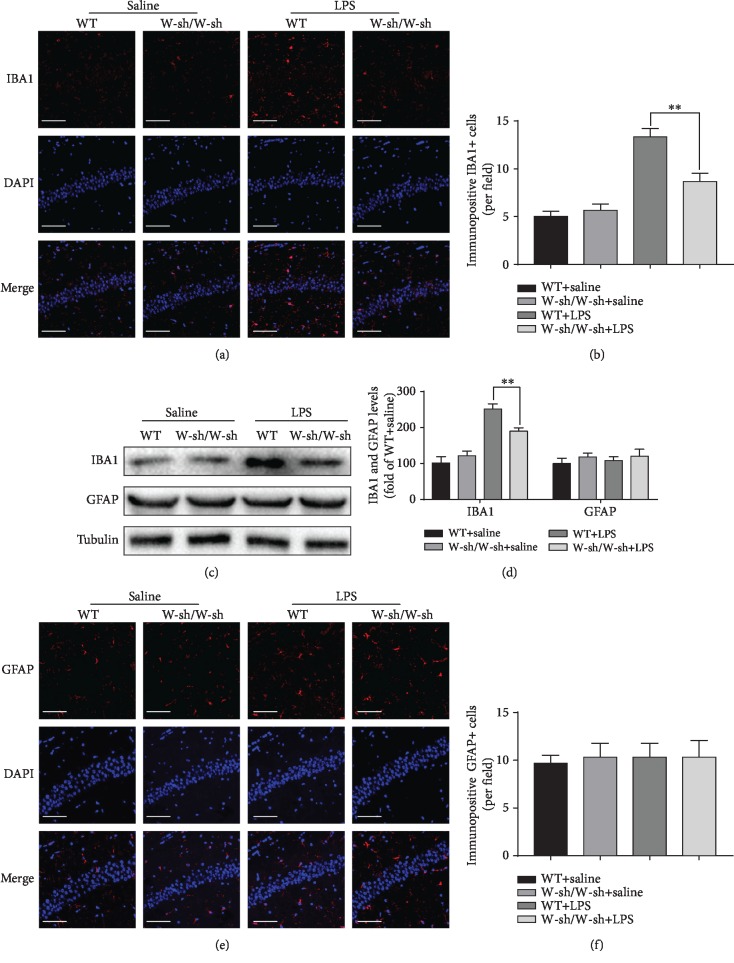
Glial changes after LPS treatment in the hippocampi of WT and Kit^W-sh/W-sh^ mice. (a) Immunofluorescence staining was used to detect ionized calcium binding adaptor molecule 1 (IBA1), a marker of microglia. Scale bar = 100 *μ*m. (b) Quantification of IBA1-positive cells in the CA1 of the hippocampus. (c) Protein levels of IBA1 and glial fibrillary acidic protein (GFAP) in the hippocampus were detected by western blotting. (d) Expression levels of IBA1 and GFAP were quantified and normalized to Tubulin levels. Each value was expressed relative to that of the WT+saline group, which was set to 100. (e) Immunofluorescence staining was used to detect GFAP, a marker of astrocytes. Scale bar = 100 *μ*m. (f) Quantification of GFAP-positive cells in the CA1 of the hippocampus. ^∗^*P* < 0.05 and ^∗∗^*P* < 0.01 vs. the WT+LPS group. Data are presented as the mean ± SEM (*n* = 6).

Overactivated microglia precede and amplify neuroinflammation by producing numerous inflammatory mediators, which induce more widespread damage to neighbor neurons. In order to investigate the effects of MCs on microglial activation, we examined the expression levels of Iba1, a typical marker for microglia, using immunofluorescence staining (Figures 6(a) and 6(b)) and western blotting (Figures 6(c) and 6(d)). No noticeable alterations of Iba1 expression were found in saline-treated WT and saline-treated Kit^W-sh/W-sh^ mice (immunofluorescent staining: adjusted *P* = 0.9433; western blotting: adjusted *P* = 0.4013). LPS administration for 4 h induced notable microglial activation in the hippocampi in WT mice (immunofluorescent staining: adjusted *P* = 0.0001; western blotting: adjusted *P* < 0.0001) and mast cell-deficient Kit^W-sh/W-sh^ mice (immunofluorescent staining: adjusted *P* = 0.0353; western blotting: adjusted *P* = 0.0014), as demonstrated by a significant increase in Iba1 protein expression. Notably, a significantly lower activation of microglia was observed in LPS-treated Kit^W-sh/W-sh^ mice than in LPS-stimulated WT mice (immunofluorescent staining: adjusted *P* = 0.0084; western blotting: adjusted *P* = 0.0026). The findings further demonstrate that MCs play a vital role in microglial overactivation and neuroinflammation.

### 3.4. LPS Treatment Has No Effects on Astrocytic Activation in the Hippocampi of WT and Kit^W-sh/W-sh^ Mice

The presence of reactive astrocytes, characterized by increased expression of the specific marker GFAP, is a hallmark of the response of the CNS to injury or inflammation. Therefore, the expression of GFAP protein (Figure 6(c)) was assessed 4 h after LPS injection by western blotting. As shown in Figure 6(d), LPS injection did not increase GFAP expression in either WT or Kit^W-sh/W-sh^ mice (*F*(3, 8) = 1.241, *P* = 0.3572). Further, we also performed immunofluorescence staining to determine changes in GFAP immunoreactivity in the hippocampus. In line with western blotting results (*F*(3, 8) = 0.05479, *P* = 0.9819), no detectable differences in GFAP immunoreactivity were observed between WT and Kit^W-sh/W-sh^ mice (Figures 6(e) and 6(f)).

### 3.5. LPS-Induced BBB Hyperpermeability Is Reduced in the Hippocampi of Kit^W-sh/W-sh^ Mice

A number of mediators (including histamine and *β*-tryptase) released by MCs may increase vascular permeability. Our previous study also demonstrated that cromolyn, a “MC stabilizer,” could improve BBB disruption induced by surgery [9]. In this study, we hypothesized that MCs play a vital role in mediating BBB damage.

**Figure 7:**
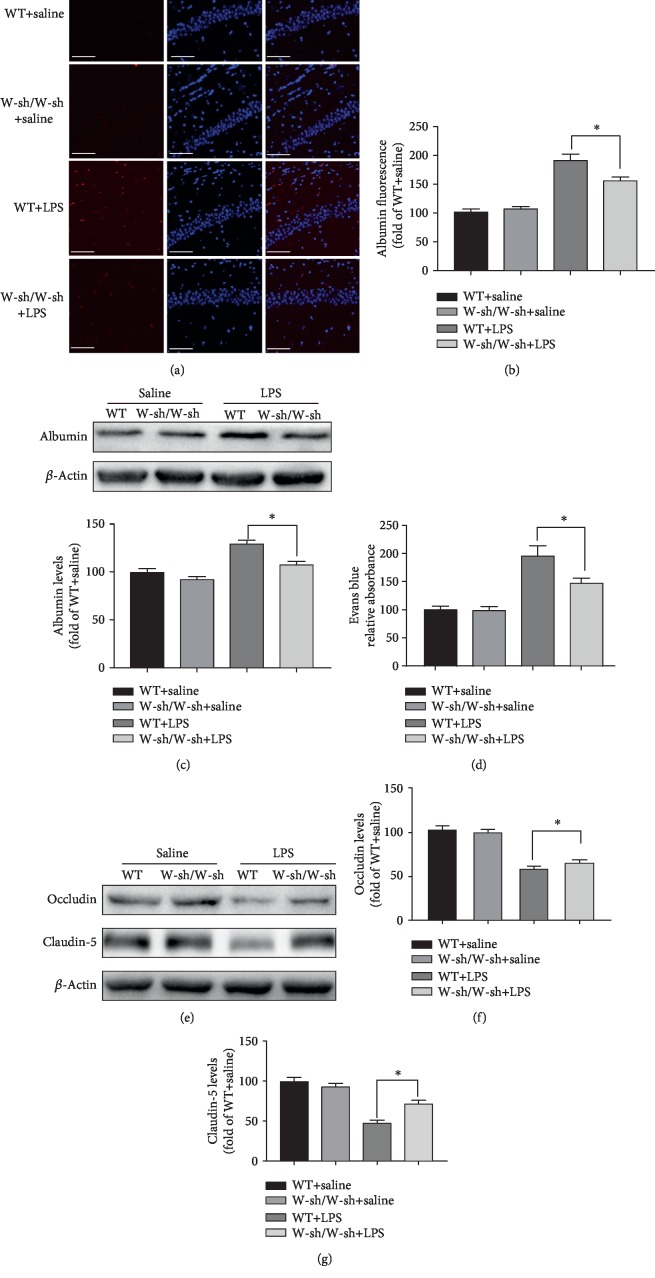
LPS-induced blood-brain barrier hyperpermeability is reduced in the hippocampi of Kit^W-sh/W-sh^ mice. (a) Representative images acquired by confocal microscopy show the albumin levels in the CA1 of the hippocampus. The arrow indicates high albumin immunoreactivity in the CA1. Scale bar = 100 mm. (b) Quantitative data of mean intensities of albumin fluorescence. (c) Protein levels of albumin were detected by western blotting. Expression levels of albumin were quantified and normalized to *β*-actin levels. (d) The quantitative analysis of Evan's blue leakage in the hippocampus. (e) Expression levels of occludin and claudin-5 were detected in the hippocampus by western blotting. (f, g) Expression levels of occludin and claudin-5 were quantified and normalized to *β*-actin levels. Each value was expressed relative to the value of the WT+saline group, which was set to 100. ^∗^*P* < 0.05 and ^∗∗^*P* < 0.01 vs. the WT+LPS group. Data are presented as the mean ± SEM (*n* = 6).

The opening of the BBB results in extravasation of serum proteins, and albumin is the most abundant serum protein. The leakage of albumin was evaluated by immunofluorescence (Figure 7(a)). The albumin immunofluorescence intensity was low in the hippocampal subfields in sections obtained from saline-exposed WT and Kit^W-sh/W-sh^ mice (immunofluorescent staining: adjusted *P* = 0.9826; western blotting: adjusted *P* = 0.2820). However, the albumin staining intensity obviously increased after 4 h of LPS exposure in WT mice (adjusted *P* < 0.0001) and Kit^W-sh/W-sh^ mice (adjusted *P* = 0.0051). Moreover, diminished staining intensity of albumin (adjusted *P* = 0.035) was found in LPS-treated Kit^W-sh/W-sh^ mice compared with LPS-treated WT mice (Figure 7(b)). Further, western blotting revealed an increase in the albumin level after LPS administration in WT mice (adjusted *P* = 0.0025) and Kit^W-sh/W-sh^ mice (adjusted *P* = 0.0344), but albumin protein expression (adjusted *P* = 0.0167) significantly decreased in LPS-challenged Kit^W-sh/W-sh^ mice compared with LPS-treated WT mice (Figure 7(c)).

EB extravasation is also a widely used marker for BBB breakage. As shown in Figure 7(d), EB levels were similar in saline-treated WT mice and saline-treated mast cell-deficient Kit^W-sh/W-sh^ mice (adjusted *P* = 0.998). A marked increase in EB levels was observed in the LPS-administrated mice compared with saline-treated mice (WT mice: adjusted *P* = 0.0005; mast cell-deficient Kit^W-sh/W-sh^ mice: adjusted *P* = 0.0336), indicating that LPS treatment induces BBB disruption. However, EB extravasation significantly decreased (adjusted *P* = 0.0238) in LPS-administrated Kit^W-sh/W-sh^ mice compared with LPS-administrated WT mice (Figure 7(d)).

Occludin and claudin-5, the integral membrane proteins consisting of tight-junction strands, contribute to BBB integrity. We next examined the expression levels of occludin and claudin-5 using western blotting. As shown in Figure 7(e), the expression of occludin (adjusted *P* = 0.9966) and occludin (adjusted *P* = 0.8) was similar in saline-treated WT mice and saline-treated mast cell-deficient Kit^W-sh/W-sh^ mice.

The protein expression levels of occludin (WT mice: adjusted *P* = 0.004; Kit^W-sh/W-sh^ mice: adjusted *P* = 0.0374) and claudin-5 (WT mice: adjusted *P* = 0.0002; Kit^W-sh/W-sh^ mice: adjusted *P* = 0.0422) were reduced 4 h after LPS challenge in WT mice and Kit^W-sh/W-sh^ mice. LPS-stimulated Kit^W-sh/W-sh^ mice showed higher protein levels of occludin (Figure 7(f); adjusted *P* = 0.0404) and claudin-5 (Figure 7(g); adjusted *P* = 0.0232) than did LPS-stimulated WT mice, and the findings are in line with those in the albumin leakage study. These results indicate that MCs may have a detrimental effect on the BBB, thus causing an increase in permeability to large molecules.

## 4. Discussion

CNS diseases affect millions of people and impose psychological burdens on their families with long-term sequelae, high rates of hospitalizations, and ongoing cognitive impairments [31]. It is widely believed that disturbances of CNS homeostasis, including injury, ischemia, infection, and neurodegenerative diseases, evoke neuroinflammatory responses in the brain [32].

MCs have a widespread distribution throughout the body, and brain MCs are highly concentrated close to blood vessels on the brain side of the BBB directly exposed to the outer environment [9, 33]. MCs act as a highly rapid response system and defend the brain from peripheral inflammation and other environmental threats, including invading pathogenic organisms. In fact, the modulatory roles of MCs in neuroinflammation have increasingly been emphasized. Adult SD rats treated with the MC stimulator, compound 48/80 (C48/80), showed increased TNF-*α* and IL-6 levels in the hypothalamus, but the increase was attenuated by treatment with the MC stabilizer cromoglycate [34, 35]. Christy et al. found that CNS infiltration of T cells was greatly attenuated in mice with MC deficiency but was fully restored upon MC reconstitution [15]. Several studies have demonstrated that MCs may work as catalysts and amplifier during neuroinflammation, potentially leading to BBB hyperpermeability [36], neuronopathy, and neurodegeneration [7, 9, 37]. However, those studies only focused on one endpoint, and the temporal changes in MC number and activation in the process of neuroinflammation are poorly understood. In the present study, we investigated the time-course changes in MC activation in the hippocampus in LPS-treated rats. A significant increase in the number of MCs was found 2 h after LPS treatment. The increased number of MCs peaked at 4 h and remained elevated through 24 h after LPS administration.

Accumulating evidence indicates that MCs are among the earliest participants in the disease-initiating events. The study by Lindsberg et al. indicates that MCs act as early responders in the regulation of acute BBB changes after cerebral ischemia and hemorrhage [17]. In addition, Jin et al. demonstrated that MCs are the first cells to respond to hypoxia-ischemia in the brain, and their recruitment and activation precede responses of neurons, glia, and endothelial cells by 2-4 hours [16]. Some studies showed that meningeal MCs, responding to danger signals, might be the “first responder” in experimental autoimmune encephalomyelitis disease [15]. In this study, we found that MC activation was prominent in the hippocampus at the early stage (0-4 h) of LPS stimulus, suggesting that MCs work as sensors during the first few hours of a proinflammatory response, at least in the LPS-induced neuroinflammation model.

Actually, it is generally accepted that circulatory MCs are committed precursors rather than mature cells, and the rapid increase of mature MCs in the brain suggests their circulation origin. Silverman et al. showed that MCs could indeed penetrate the CNS [38]. Besides, MCs can adhere to endothelia and exhibit rolling behavior in a P-selectin-dependent manner [39]. In detail, MCs could induce upregulation of P-selectin and intercellular adhesion molecule 1 on carotid endothelial cells through a histamine-independent mechanism [40]. In addition, the chemokine receptor CXCR4 is expressed within the MC lineage and its ligand stromal cell-derived factor-1*α* acts as a MC chemotaxin [41]. Dudeck et al. demonstrated that MCs could migrate across an endothelial barrier in response to TNF predominantly mediated by PECAM-1 and VCAM-1 [42]. MCs also contain many proteases, such as *β*-tryptase, which may create a pathway through the endothelial junctions and the extracellular matrix of the basal lamina. We have observed that the disruption of BBB may initiate at 1~2 h after LPS treatment, as determined by albumin leakage (Supplemental [Supplementary-material supplementary-material-1]) and occludin and claudin-5 protein changes (Supplemental [Supplementary-material supplementary-material-1]) in the brain. However, the mechanism whereby the MCs transit the brain capillary endothelium and its basal lamina is poorly understood and remains to be characterized in detail. This has urged the need for new studies to help understand how MCs interact with BBB to trigger neuroinflammation, to what extent cell-to-cell contact is required for such interaction, and how different MC mediators contribute to such communication. These processes may reveal opportunities to inhibit neuroinflammation by modulating peripheral tissue numbers of MCs.

MCs are considered one of the fastest responders due to their ability to rapidly release prestored and newly synthesized mediators in their cytoplasmic secretory granules after activation [43]. Various immunomodulatory compounds, including *β*-tryptases [29] and histamine [30], are significant constituents of mature MC granules. In this study, the levels of histamine and *β*-tryptase in the hippocampus markedly and rapidly increased at 6 h after LPS treatment and then remained higher than the baseline levels within 24 h. These changes occurred just after MC activation, suggesting that MCs can quickly modulate the levels of histamine and *β*-tryptase.

Studies showed that MC *β*-tryptase could activate proteinase-activated receptor 2 receptors, which contribute to the degradation of tight junction proteins, in brain microvascular endothelial cells [20]. Furthermore, microglia express all four histamine receptors (H1R, H2R, H3R, and H4R), and stimulation of mitogen-activated protein kinases (MAPK), PI3K/AKT, and nuclear factor kappa B (NF-kappa B) signaling pathways through H1R and H4R leads to the production of TNF-*α* and IL-6 [19, 34, 35]. These functional interactions highly suggest communications of MCs with microglia, and BBB might orchestrate the whole inflammatory process. In this study, we treated mast cell-deficient Kit^W-sh/W-sh^ mice with LPS for 4 h to induce neuroinflammation. Our findings demonstrated that BBB disruption was significantly inhibited in LPS-treated mast cell-deficient Kit^W-sh/W-sh^ mice compared with LPS-treated WT mice. Consistently, the cytokine secretion and microglia activation were significantly reduced in Kit^W-sh/W-sh^ mice compared with WT mice after LPS treatment. Therefore, the present findings further confirm the interaction of MCs, microglia, and BBB in neuroinflammation [44].

*In vitro* studies indicate that the activation of MCs cocultured with astrocytes induces production of cytokines and chemokines, including TNF-*α*, IL-6, and MCP-1 [45]. Astrocytes express histamine receptors (H1R, H2R, and H3R) [46], indicating MCs may also regulate astrocytic responses. Therefore, considering their common perivascular localization, potential communication between astrocytes and MCs might exist. In fact, pretreatment with the “MC stabilizer” cromolyn inhibits surgery-induced astrocyte activation at 1 day after the operation [10]. However, no detectable astrocytic changes were found in WT and Kit^W-sh/W-sh^ mice at 4 h after LPS stimulation, which might be owing to the fact that the activation of astrocytes lags behind the microglia [47–49]. Moreover, Norden et al. also found that rapid microglial cytokine induction preceded astrocytic cytokine expression after acute LPS administration [22]. Our preliminary study also indicated the astrocytes activated until 24 h after LPS stimulation. The data showed LPS treatment for 12 h did not increase GFAP expression in either WT or Kit^W-sh/W-sh^ mice, while LPS administration for 24 h induced astrocytic activation in the hippocampi in WT mice and mast cell-deficient Kit^W-sh/W-sh^ mice, as demonstrated by a significant increase in GFAP expression. Notably, a significantly lower activation of astrocytes was observed in LPS-treated Kit^W-sh/W-sh^ mice than in LPS-stimulated WT mice (Supplemental [Supplementary-material supplementary-material-1]). Therefore, further studies are needed to further elucidate the relationship between MCs and astrocytes, as well as its role in LPS-induced neuroinflammation. The findings might significantly contribute to the development of treatment strategies of inflammation-related CNS diseases.

## 5. Conclusions

In summary, the present findings indicate that MCs participate in the early neuroinflammation through the release of proinflammatory mediators. Microglial activation, production of inflammatory factors, and BBB damage were attenuated in Kit^W-sh/W-sh^ mast cell-deficient mice 4 h after LPS treatment. Thus, MCs may have essential roles in initiating the inflammatory cascade, in particular, by serving as transducers of harmful signals to the brain. These results suggest that MCs are significant effector cells in neuroinflammatory disorders. Our findings may shed light on the new immunotherapy strategy that could complement the current treatment regimens based solely on altering the glial response.

## Data Availability

The data used to support the findings of this study are available from the corresponding authors upon request.

## Supplementary Materials

Supplementary Materials Supplemental Figure 1: blood-brain barrier permeability changes in the hippocampi of rats after LPS treatment. A. The expression of albumin, occludin, and claudin-5 was detected in the hippocampi of rats by western blotting. B-D. The expression of albumin, occludin, and claudin-5 was quantified and normalized to *β*-actin levels. Each value is expressed relative to that in the control group, which was set to 100. ^∗^*P* < 0.05 and ^∗∗^*P* < 0.01 vs. controls. Data are presented as the mean ± SEM (*n* = 6). Supplemental Figure 2: astrocytic changes after 12 h and 24 h treatment of LPS in the hippocampi in WT and Kit^W-sh/W-sh^ mice. A. Protein levels of GFAP in the hippocampus were detected by western blotting. B. Expression levels of GFAP were quantified and normalized to Tubulin levels. Each value was expressed relative to that of the W+saline group, which was set to 100. C. Immunofluorescence staining was used to detect GFAP, a marker of astrocytes. Scale bar = 100 *μ*m. D. Quantification of GFAP-positive cells in the CA1 of the hippocampus. ^∗^*P* < 0.05 and ^∗∗^*P* < 0.01 vs. the WT+saline group. ^#^*P* < 0.05 and ^##^*P* < 0.01 vs. the WT+LPS-24 h group. Data are presented as the mean ± SEM (*n* = 6). Supplemental Figure 3: Fc*ε*RI is not expressed on MCs in the hippocampi of SD rats by immunofluorescence labeling (*n* = 4). Scale bar = 100 *μ*m.
