# Supplementary material for: The Mast Cell Is an Early Activator of Lipopolysaccharide-Induced Neuroinflammation and Blood-Brain Barrier Dysfunction in the Hippocampus
Source: Mediators Inflamm. 2020 Feb 24;2020:8098439. doi: 10.1155/2020/8098439 (PMC7060448; doi:10.1155/2020/8098439)
Supplement: Supplementary Materials — Supplemental Figure 1: blood-brain barrier permeability changes in the hippocampi of rats after LPS treatment. A. The expression of albumin, occludin, and claudin-5 was detected in the hippocampi of rats by western blotting. B-D. The expression of albumin, occludin, and claudin-5 was quantified and normalized to β-actin levels. Each value is expressed relative to that in the control group, which was set to 100. ∗P < 0.05 and ∗∗P < 0.01 vs. controls. Data are presented as the mean ± SEM (n = 6). Supplemental Figure 2: astrocytic changes after 12 h and 24 h treatment of LPS in the hippocampi in WT and KitW-sh/W-sh mice. A. Protein levels of GFAP in the hippocampus were detected by western blotting. B. Expression levels of GFAP were quantified and normalized to Tubulin levels. Each value was expressed relative to that of the W+saline group, which was set to 100. C. Immunofluorescence staining was used to detect GFAP, a marker of astrocytes. Scale bar = 100 μm. D. Quantification of GFAP-positive cells in the CA1 of the hippocampus. ∗P < 0.05 and ∗∗P < 0.01 vs. the WT+saline group. #P < 0.05 and ##P < 0.01 vs. the WT+LPS-24 h group. Data are presented as the mean ± SEM (n = 6). Supplemental Figure 3: FcεRI is not expressed on MCs in the hippocampi of SD rats by immunofluorescence labeling (n = 4). Scale bar = 100 μm. [file 8098439.f1.pdf]

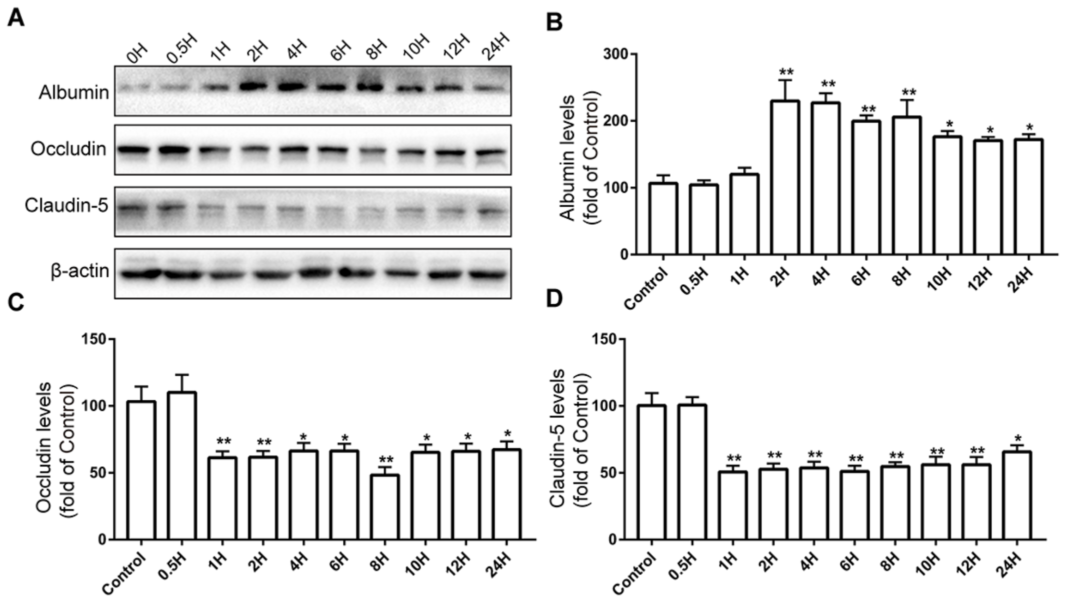

Supplemental figure 1. Blood-brain barrier permeability changes in the hippocampi of rats after LPS treatment. **A.** The expression of albumin, occludin and claudin-5 was detected in the hippocampi of rats by western blotting. **B-D.** The expression of albumin, occludin and claudin-5 was quantified and normalized to β-actin levels. Each value is expressed relative to that in the control group, which was set to 100. $^{*}P < 0.05$, $^{**}P < 0.01$ vs. controls. Data are presented as mean ± SEM (n = 6).

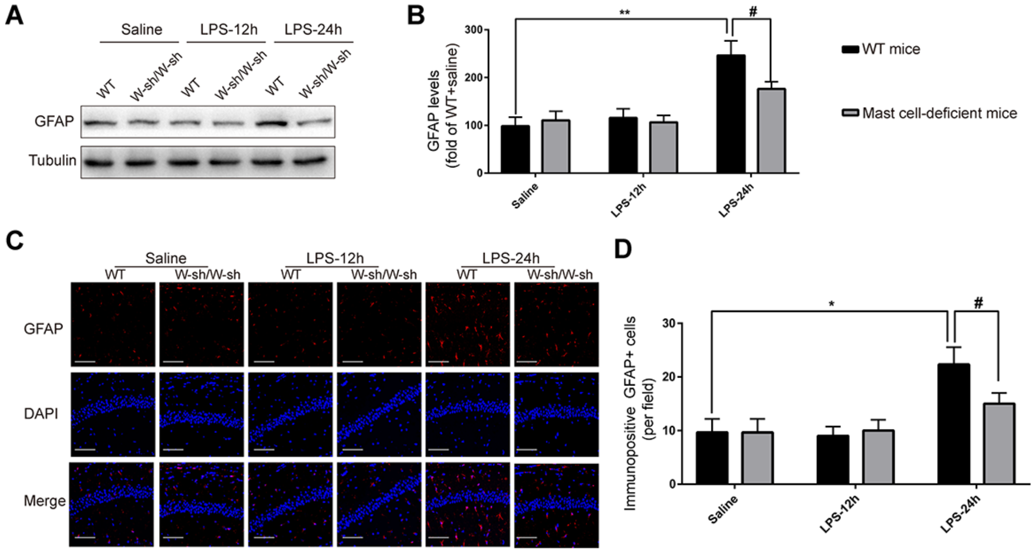

Supplemental figure 2. Astrocytic changes after 12h and 24 h treatment of LPS in the hippocampi in WT and $Kit^{W\text{-}sh/W\text{-}sh}$ mice. **A.** Protein levels of GFAP in the hippocampus were detected by Western blotting. **B.** Expression levels of GFAP were quantified and normalized to Tubulin levels. Each value was expressed relative to that of the WT + saline group, which was set to 100. **C.** Immunofluorescence staining was used to detect GFAP, a marker of astrocytes. Scale bar = 100 μm. **D.** Quantification of GFAP-positive cells in the CA1 of the hippocampus. $^{*}P < 0.05$, $^{**}P < 0.01$ vs. the WT + saline group. $^{\#}P < 0.05$, $^{\#\#}P < 0.01$ vs. the WT+LPS-24h group. Data are presented as mean ± SEM (n = 6).

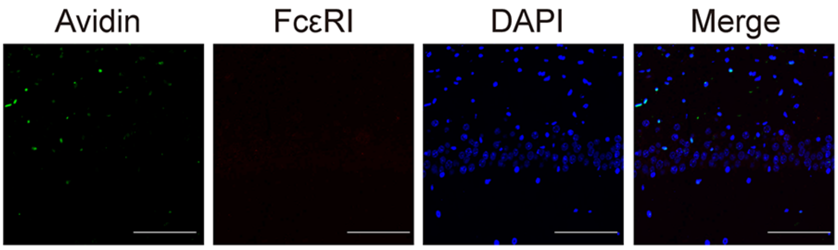

Supplemental figure 3. FcεRI is not expressed on MCs in the hippocampi of SD rats by immunofluorescence labeling (n=4). Scale bar, 100 μm.
